# Supplementary material for: Prognostic significance of PI3K/AKT/ mTOR signaling pathway members in clear cell renal cell carcinoma
Source: PeerJ. 2020 Jun 1;8:e9261. doi: 10.7717/peerj.9261 (PMC7271881; doi:10.7717/peerj.9261)
Supplement: Table S1 [file peerj-08-9261-s001.doc]

**Supplementary Table 1. GEPIA box plots of the mRNA expression profiles of PI3K/AKT/mTOR signaling pathway proteins in 523 clear cell renal cell carcinoma (T) and 72 normal kidney tissues (N), which was downloaded from TCGA database.** **
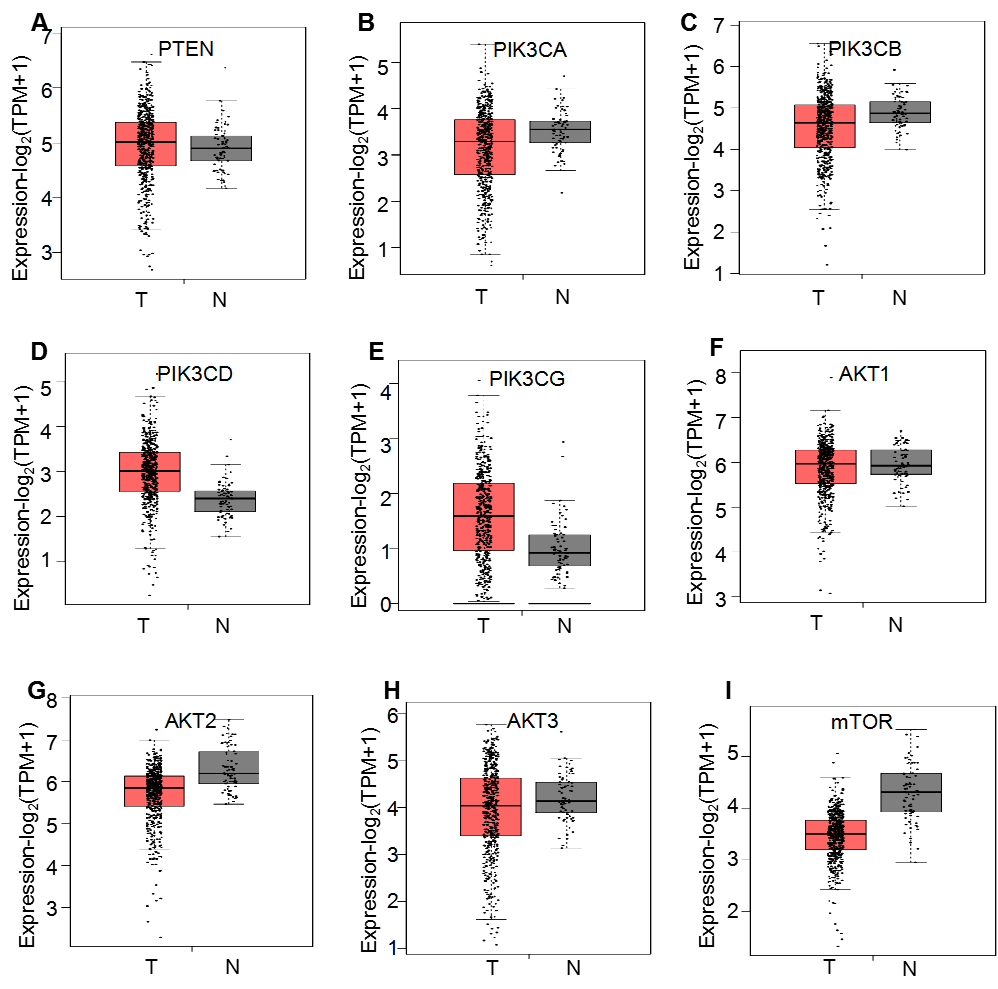
**

(A-I) Box plots demonstrate the relative mRNA expression levels (log2 (TPM+1)) of (A) *PTEN*, (B) *PIK3CA*, (C) *PIK3CB*, (D) *PIK3CD*, (E) *PIK3CG*, (F) *AKT1*, (G) *AKT2*, (H) *AKT3* and (I) *mTOR,* which were not significantly altered between the tissues (P>0.05).
