# Supplementary material for: Prognostic significance of PI3K/AKT/ mTOR signaling pathway members in clear cell renal cell carcinoma
Source: PeerJ. 2020 Jun 1;8:e9261. doi: 10.7717/peerj.9261 (PMC7271881; doi:10.7717/peerj.9261)
Supplement: Table S2 [file peerj-08-9261-s002.doc]

**Supplementary Table 2. Oncomine box plots of the mRNA expression profiles of PI3K/AKT/mTOR pathway members in 10 clear cell renal cell carcinoma (T) and 10 matched normal kidney tissues (N).**


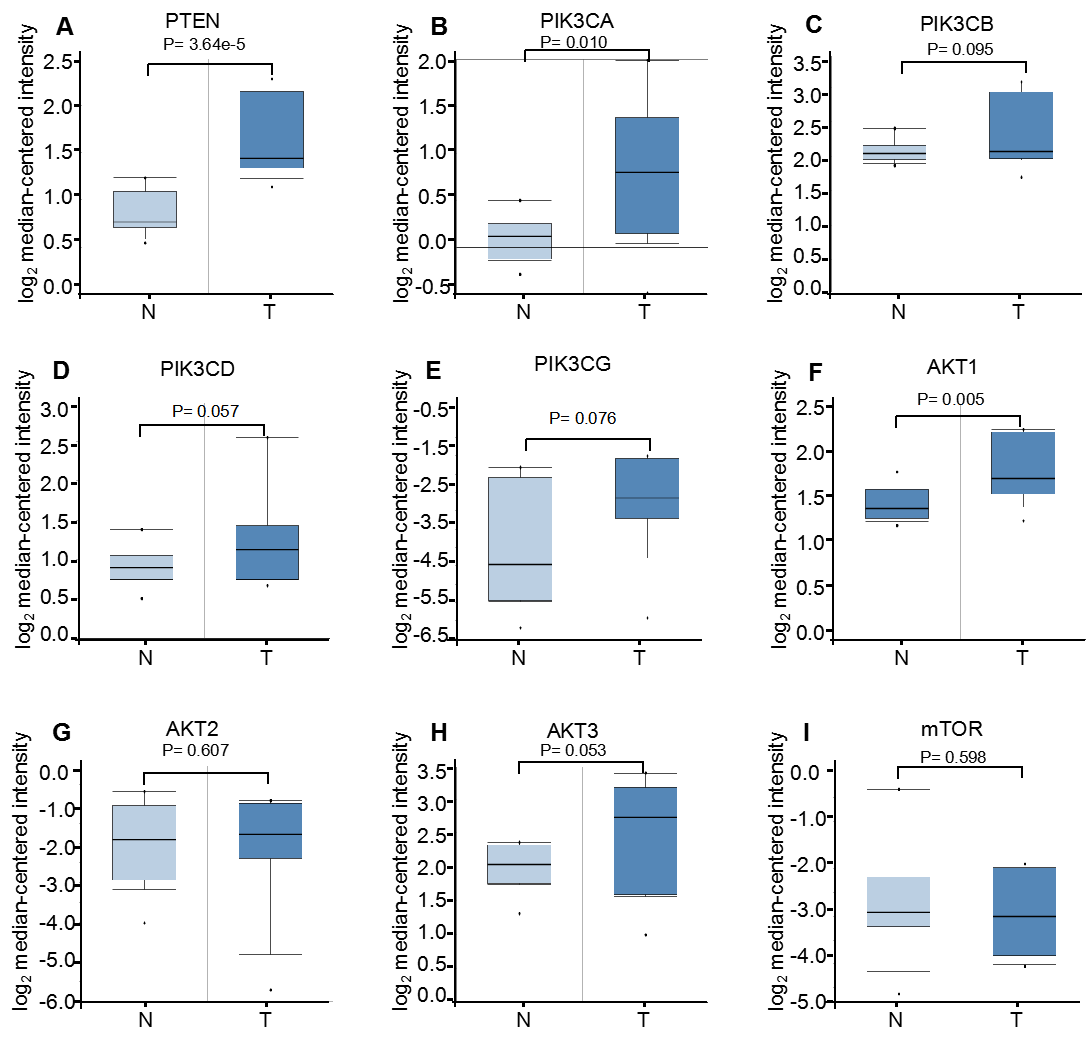


1. **I)** Box plots demonstrate the relative mRNA expression levels of (A) *PTEN*, (B) *PIK3CA*, (C) *PIK3CB*, (D) *PIK3CD*, (E) *PIK3CG*, (F) *AKT1*, (G) *AKT2*, (H) *AKT3* and (I) *mTOR,* which showed that only PTEN (1.77-fold), PIK3CA (1.56-fold) and AKT1 (1.27-fold) were increased in ccRCC, while the other six genes were not significantly altered between the tissues (P>0.05)**.**
