# Supplementary material for: Prognostic significance of PI3K/AKT/ mTOR signaling pathway members in clear cell renal cell carcinoma
Source: PeerJ. 2020 Jun 1;8:e9261. doi: 10.7717/peerj.9261 (PMC7271881; doi:10.7717/peerj.9261)
Supplement: Table S3 [file peerj-08-9261-s003.doc]

**Supplementary Table 3. Correlation of the PI3K/AKT/mTOR mRNA expression with OS in different pathological grade of ccRCC patients (K-M plotter, n=530).**

*P-*value

HR (95% CI)

n ^1^

Grade

Gene

14

227

206

75

14

227

206

75

14

227

206

75

14

227

206

75

14

227

206

75

14

227

206

75

14

227

206

75

14

227

206

75

14

227

206

75

# 2

0.51 (0.28-0.93)

0.45(0.29-0.72)

0.68 (0.38-1.25)

# 2

0.42 (0.20-0.87)

0.41 (0.26-0.65)

1.26(0.71-2.24)

# 2

1.79 (0.97-3.31)

0.34 (0.21-0.55)

0.53 (0.31-0.92)

# 2

0.52(0.28-0.98)

0.20 (0.72-1.99)

2.00 (1.00-4.00)

# 2

0.57 (0.31-1.07)

0.40 (0.25-0.64)

0.14 (0.31-0.95)

# 2

0.68 (0.37-1.24)

1.07(0.68-1.70)

1.99 (1.16-3.43)

# 2

1.07 (0.59-1.94)

1.25 (0.79-1.98)

1.29 (0.76-2.20)

# 2

0.60 (0.33-1.10)

0.59 (0.37-0.94)

0.54 (0.31-0.93)

# 2

0.70(0.38-1.28)

0.58 (0.36-0.93)

1.66 (0.96-2.87)

0.024

<0.001

0.210

0.016

<0.001

0.430

0.061

0.001

0.023

0.038

0.490

0.046

0.076

<0.001

0.031

0.200

0.760

0.011

0.810

0.340

0.340

0.093

0.025

0.025

0.24

0.023

0.069

I

Ⅱ

Ⅲ

Ⅳ

I

Ⅱ

Ⅲ

Ⅳ

I

Ⅱ

Ⅲ

Ⅳ

I

Ⅱ

Ⅲ

Ⅳ

I

Ⅱ

Ⅲ

Ⅳ

I

Ⅱ

Ⅲ

Ⅳ

I

Ⅱ

Ⅲ

Ⅳ

I

Ⅱ

Ⅲ

Ⅳ

I

Ⅱ

Ⅲ

Ⅳ

PTEN

PIK3CA

PIK3CB

PIK3CD

PIK3CG

AKT1

AKT2

AKT3

mTOR

Note: 1. The total number was 522, because there were missing expression values and/ or incomplete survival data,

1. ^#^ Samples number too low for meaningful analysis.
