# Supplementary material for: Prognostic significance of PI3K/AKT/ mTOR signaling pathway members in clear cell renal cell carcinoma
Source: PeerJ. 2020 Jun 1;8:e9261. doi: 10.7717/peerj.9261 (PMC7271881; doi:10.7717/peerj.9261)
Supplement: Dataset S1 [file peerj-08-9261-s004.docx]

**Supplementary file 1: The raw data of WB.**

| Name | Molecular weight | Original bands/gels  M N1 T1 N2 T2 N3 T3 N2 T2 |
| --- | --- | --- |
| PI3K | 110kDa  100 | 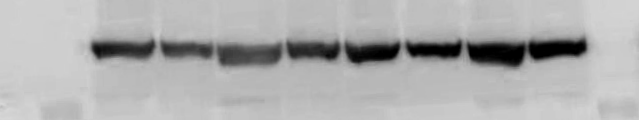 |
| Akt | 60 kDa  70 | 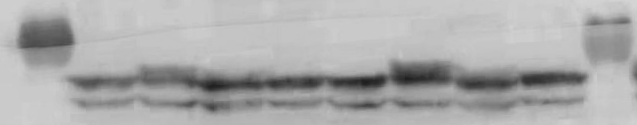 |
| mTOR | 289kDa | 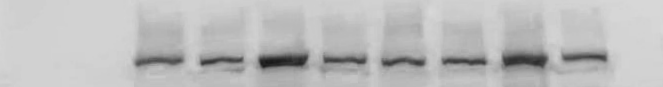 |
| Actin | 43kDa  40 | 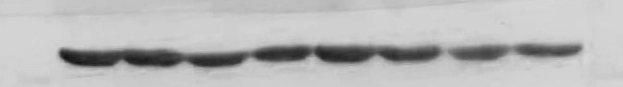 |

Note: 1. The blots with same amount of protein, i.e., PI3K, Akt, mTOR and Actin.

1. Abbreviation. N: normal kidney tissue, T: ccRCC; M: Marker.
