# Supplementary figures and images for: Prognostic significance of PI3K/AKT/ mTOR signaling pathway members in clear cell renal cell carcinoma
Source: PeerJ. 2020 Jun 1;8:e9261. doi: 10.7717/peerj.9261 (PMC7271881; doi:10.7717/peerj.9261)

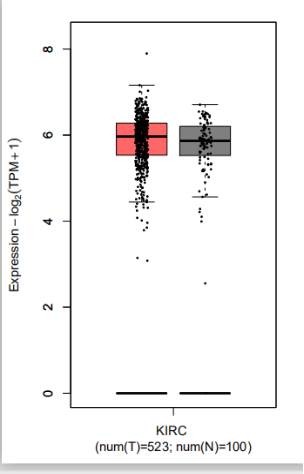

Supplement: Dataset S3 [file peerj-08-9261-s006.zip › AKT1 Gepia.png]

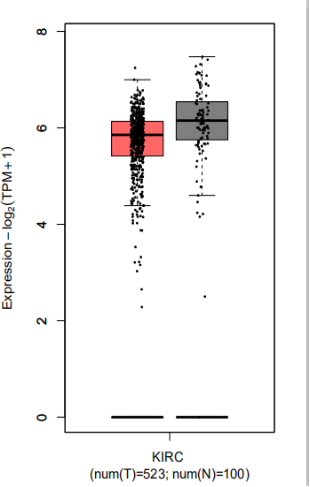

Supplement: Dataset S3 [file peerj-08-9261-s006.zip › AKT2 Gepia.png]

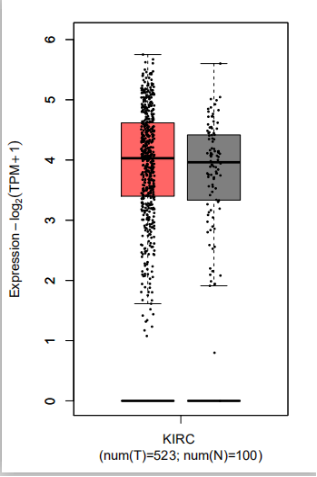

Supplement: Dataset S3 [file peerj-08-9261-s006.zip › AKT3 Gepia.png]

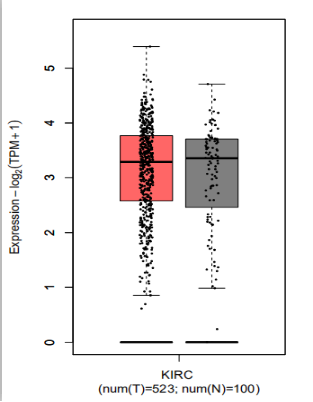

Supplement: Dataset S3 [file peerj-08-9261-s006.zip › PIK3CA Gepia.png]

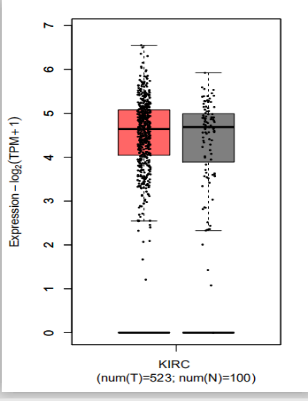

Supplement: Dataset S3 [file peerj-08-9261-s006.zip › PIK3CB Gepia.png]

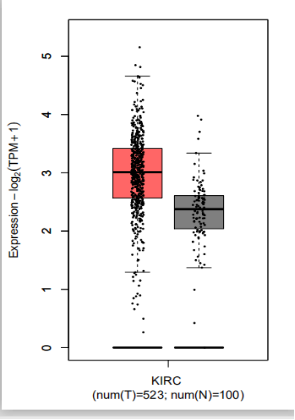

Supplement: Dataset S3 [file peerj-08-9261-s006.zip › PIK3CD Gepia.png]

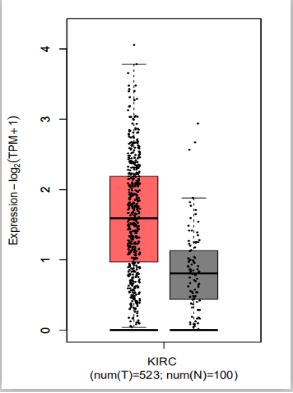

Supplement: Dataset S3 [file peerj-08-9261-s006.zip › PIK3CG Gepia.png]

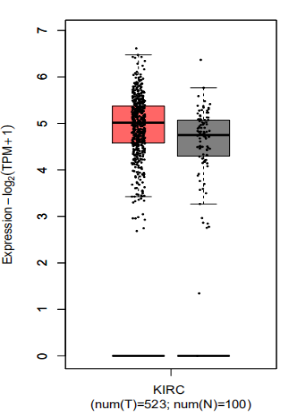

Supplement: Dataset S3 [file peerj-08-9261-s006.zip › PTEN Gepia.png]

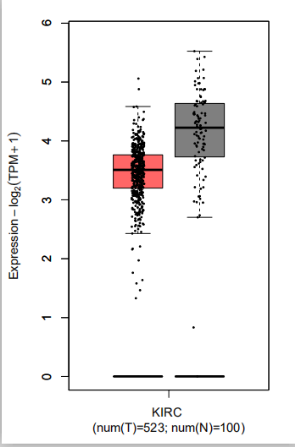

Supplement: Dataset S3 [file peerj-08-9261-s006.zip › mTOR Gepia.png]

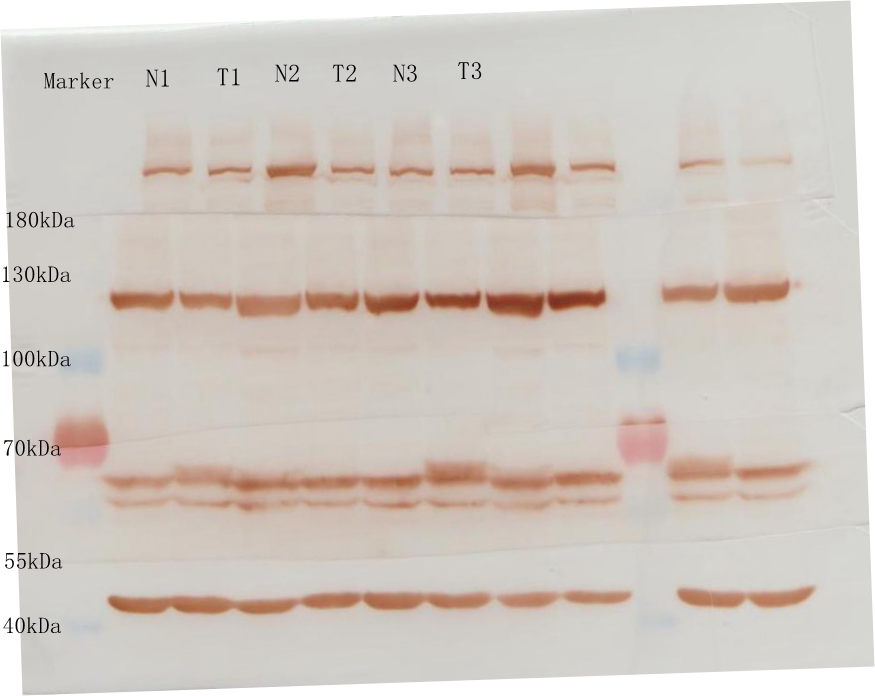

Supplement: Dataset S4 [file peerj-08-9261-s007.zip › mTOR1_original_╕▒▒╛.tiff]

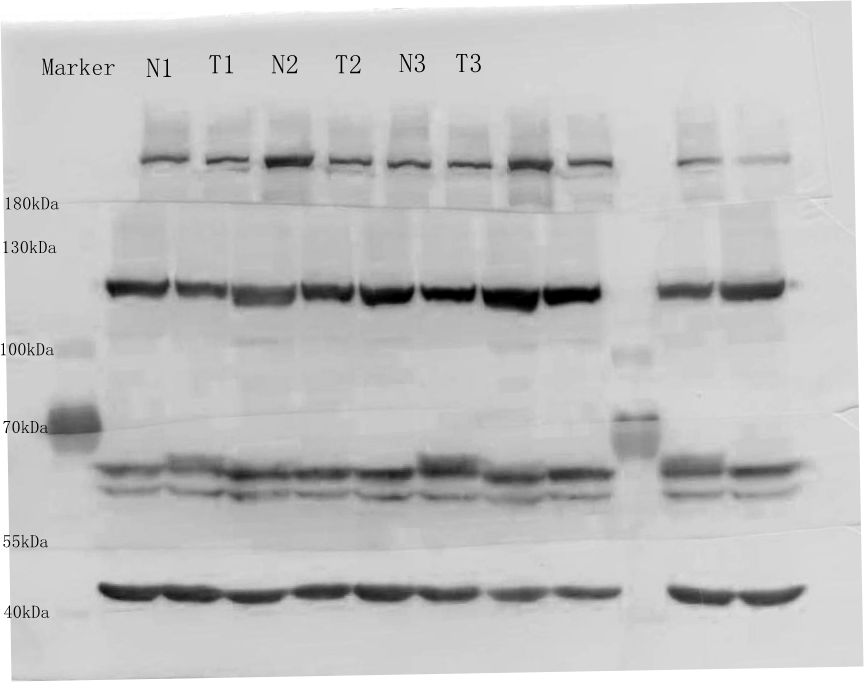

Supplement: Dataset S4 [file peerj-08-9261-s007.zip › mTOR1_black_╕▒▒╛.tiff]
